# Molecularly Engineered Aza-Crown Ether Functionalized Sodium Alginate Aerogels for Highly Selective and Sustainable Cu^2+^ Removal

**DOI:** 10.3390/gels12010078

**Published:** 2026-01-16

**Authors:** Teng Long, Ayoub El Idrissi, Lin Fu, Yufan Liu, Banlian Ruan, Minghong Ma, Zhongxun Li, Lingbin Lu

**Affiliations:** 1 School of Materials Science and Engineering, Hainan University, Haikou 570228, China; lonteg@hainanu.edu.cn (T.L.); ayoub.elidrissi@hainanu.edu.cn (A.E.I.); fulin@hainanu.edu.cn (L.F.);; 2 State Key Laboratory of Tropic Ocean Engineering Materials and Materials Evaluation, Haikou 570228, China; 3 Special Glass Key Lab of Hainan Province, Haikou 570228, China

**Keywords:** sodium alginate, aza-crown ether, aerogel, copper removal, high selectivity

## Abstract

Developing sustainable and molecularly selective adsorbents for heavy-metal removal remains a critical challenge in water purification. Herein, we report a green molecular-engineering approach for fabricating aza-crown ether functionalized sodium alginate aerogels (ACSA) capable of highly selective Cu^2+^ capture. The aerogels were synthesized via saccharide-ring oxidation, Cu^2+^-templated self-assembly, and reductive amination, enabling the covalent integration of aza-crown ether motifs within a hierarchically porous biopolymer matrix. Structural analyses (FTIR, ^13^C NMR, XPS, SEM, TGA) confirmed the in situ formation of macrocyclic N/O coordination sites. Owing to their interconnected porosity and chemically stable framework, ACSA exhibited rapid sorption kinetics following a pseudo-second-order model (R^2^ = 0.999) and a Langmuir maximum adsorption capacity of 150.82 mg·g^−1^. The material displayed remarkable Cu^2+^ selectivity over Zn^2+^, Cd^2+^, and Ni^2+^, arising from the precise alignment between Cu^2+^ ionic radius (0.73 Å) and crown-cavity dimensions, synergistic N/O chelation, and Jahn-Teller stabilization. Over four regeneration cycles, ACSA retained more than 80% of its original adsorption capacity, confirming excellent durability and reusability. This saccharide-ring modification strategy eliminates crown-ether leaching and weak anchoring, offering a scalable and environmentally benign route to bio-based adsorbents that combine molecular recognition with structural stability for efficient Cu^2+^ remediation and beyond.

## 1. Introduction

The persistent release of heavy metals from industrial and urban activities poses a serious threat to aquatic ecosystems and human health [1]. Among them, copper ions (Cu^2+^) are of particular concern because of their dual biological and toxic roles. While trace Cu^2+^ is essential for enzymatic and metabolic processes, excessive accumulation disrupts redox balance, damages cellular components, and causes severe hepatic and neurological disorders [1,2,3]. The continuous discharge of Cu^2+^-rich effluents from electroplating, metallurgy, and electronics manufacturing has therefore intensified the demand for efficient, selective, and sustainable copper-removal technologies [4]. Conventional treatment technologies, including chemical precipitation, ion exchange, membrane separation, and electrochemical oxidation, have been extensively used for heavy-metal remediation. However, these methods often suffer from high operational costs, energy intensity, secondary sludge generation, and insufficient metal selectivity [5,6]. Adsorption has therefore emerged as one of the most promising alternatives owing to its simplicity, scalability, cost-effectiveness, and ability to efficiently remove trace contaminants [7]. The performance of adsorption, however, critically depends on the design of the adsorbent, including its surface chemistry, porosity, and binding affinity toward specific ions [8,9,10]. Hence, developing functional adsorbents with selective recognition sites, robust architectures, and environmental compatibility remains a central challenge in water treatment materials.

Traditional inorganic adsorbents such as activated carbon, zeolites, and metal oxides exhibit high surface area and structural stability but lack tunable functional groups and display limited selectivity toward specific metal ions. Metal–organic frameworks (MOFs) and covalent organic frameworks (COFs) offer tailored porosity and tunable coordination chemistry, yet their high synthesis cost, poor chemical stability in aqueous media, and difficulty in recovery impede large-scale applications [11]. Polymeric and biopolymeric adsorbents, on the other hand, provide flexibility, processability, and biocompatibility, but their limited binding-site density and poor recyclability restrict their overall efficiency [12]. Consequently, the rational design of green adsorbents that integrate molecular-level selectivity, mechanical durability, and sustainability is urgently required.

In this context, crown ethers have emerged as a prominent class of molecular receptors because their preorganized macrocyclic cavities and multidentate donor arrays enable highly efficient and selective ion recognition [13]. Selectivity is largely dictated by cavity–ion complementarity: tailoring the ring size to the target cation promotes preferential complexation through synergistic electrostatic and coordination interactions [14,15]. This concept is exemplified by benzo-crown ethers, where benzo-18-crown-6 (B18C6) and benzo-21-crown-7 (B21C7) display strong affinities toward Sr^2+^ and Cs^+^, respectively. The cavity of B18C6 (2.6–3.2 Å) is well aligned with the ionic radius of Sr^2+^ (1.18 Å), whereas the larger cavity of B21C7 (3.4–4.0 Å) better accommodates Cs^+^ (3.29 Å), thereby strengthening host-guest binding via oxygen coordination coupled with π-donor contributions [16]. Despite these advantages, conventional crown-ether-based liquid–liquid extraction remains limited by solvent toxicity and operational complexity, constraining scalability and environmental compatibility. In parallel, the direct deployment of free crown ethers in aqueous systems is often undermined by toxicity concerns, poor water compatibility, and receptor leaching, which erode both safety and long-term performance. Accordingly, immobilization of crown ethers within solid matrices is essential to translate their molecular recognition into practical, reusable, and environmentally responsible separation and remediation platforms.

Recent literature provides clear evidence that immobilization can unlock both high uptake and operational stability. Jing et al. [17] developed an anisotropic [2,4]-dibenzo-18-crown-6 grafted bamboo-pulp aerogel featuring directional channels, ultrahigh porosity (~97.67%) and a large specific surface area (~103.7 m^2^·g^−1^), and reported maximum adsorption capacities of 129.15 mg·g^−1^ (Pb^2+^), 29.85 mg·g^−1^ (Cu^2+^), and 27.89 mg·g^−1^ (Cd^2+^), while maintaining regenerability over 5 cycles highlighting how porous aerogels can preserve accessibility while stabilizing crown-ether sites. Furthermore, Zhou et al. [16] reported a dual crown-ether modified chitosan (CTS/B18C6/B21C7) designed for “crown-position recognition”, achieving maximum adsorption capacities of 172.11 mg·g^−1^ (Cs^+^) and 132.23 mg·g^−1^ (Sr^2+^), demonstrating that cooperative cavity matching can be effectively translated into solid sorbents for selective radionuclide capture. In a membrane format, Du et al. [18] fabricated crown-ether grafted polyimide nanofiber membranes via in situ grafting and electrospinning; the optimized membrane delivered a Cs^+^ adsorption capacity of 85.23 mg·g^−1^, substantially higher than the ungrafted control (68.21 mg·g^−1^), confirming the role of covalently introduced crown-ether sites in boosting both capacity and selectivity in a processable architecture. Finally, Supraja and Karpagam [19] embedded pyridine-linked crown ethers into PVDF to form composite membranes and reported adsorption capacities of 42.36 mg·g^−1^ (Cu^2+^) and 68.97 mg·g^−1^ (Ag^+^) for PVDF-CEPY-1 under optimal conditions (pH 5, 298 K), with >85% capacity retained after 10 cycles, underscoring the importance of immobilization for durability and reuse. Accordingly, immobilizing crown ethers within solid matrices is essential to translate their molecular recognition into practical, reusable, and environmentally responsible separation platforms. Building on this rationale, 15-crown-5 is particularly attractive for copper capture because its cavity diameter (~1.7–2.2 Å) closely matches the ionic radius of Cu^2+^ (~1.9 Å), enabling selective chelation primarily through oxygen coordination [20,21]. Moreover, partial replacement of oxygen donors by nitrogen atoms affords aza-crown ethers, which typically exhibit stronger complexation toward transition-metal ions due to synergistic N/O coordination and enhanced electron-donating capability [22]. Embedding such aza-crown motifs within a porous, biocompatible host can therefore couple molecular-level selectivity with macroscopic robustness, offering a route to simultaneously achieve high Cu^2+^ affinity, minimized leaching, and sustained performance—an integration that remains challenging for many conventional adsorbents.

Sodium alginate provides an ideal, sustainable matrix to implement this concept. As a natural anionic polysaccharide extracted from brown seaweeds, alginate consists of β-D-mannuronic and α-L-guluronic acid units and contains abundant hydroxyl and carboxyl groups that enable straightforward chemical modification and additional metal coordination [23]. Importantly, alginate can be shaped into hydrogels and aerogels with interconnected porous networks and low density, while maintaining excellent biocompatibility [24,25]. When combined with chemical crosslinking to stabilize the 3D framework, alginate-based matrices can immobilize functional receptors in a mechanically robust architecture and preserve accessible transport pathways for aqueous ions. Together, these features make chemically crosslinked alginate a compelling platform for constructing green, high-performance adsorbents that integrate aza-crown ether recognition with hierarchical porosity and operational stability.

Herein, we report a facile and green synthesis strategy for the in situ construction of aza-crown ether-functionalized sodium alginate aerogels (ACSA) through a controlled saccharide-ring modification process. In this approach, sodium alginate is first oxidized using sodium periodate, which cleaves vicinal diols to generate dialdehyde groups along the polymer backbone. These aldehyde sites react with 1,2-bis(2-aminoethoxy)ethane (BAEE) in the presence of Cu^2+^ ions, which serve as a templating agent to direct the self-assembly of aza-crown ether rings via intramolecular condensation. Subsequent reductive amination stabilizes the formed macrocyclic structures, producing a three-dimensional aerogel network containing uniformly distributed aza-crown cavities covalently bound to the alginate framework. This approach addresses limitations at three levels: (i) compared with free crown ethers, it avoids dispersing small molecular receptors in water, reducing handling/leaching concerns and enabling easy separation and reuse; (ii) compared with immobilized crown ethers made by post-loading or surface grafting pre-formed macrocycles, it prevents receptor localization only on the outer surface, weak attachment, and pore blockage, because the binding cavities are generated in situ throughout the scaffold and are non-leachable; and (iii) compared with oxygen-only crown ethers, the aza-crown architecture introduces N donors that strengthen Cu^2+^ binding via synergistic N/O coordination. As a result, ACSA couples molecular-scale Cu^2+^ recognition with hierarchical aerogel porosity, improving Cu^2+^ selectivity, uptake efficiency, and cycling stability in a renewable, polysaccharide-based adsorbent platform.

## 2. Results and Discussion

### 2.1. Preparation and Characterization of ACSA

**Figure 1:**
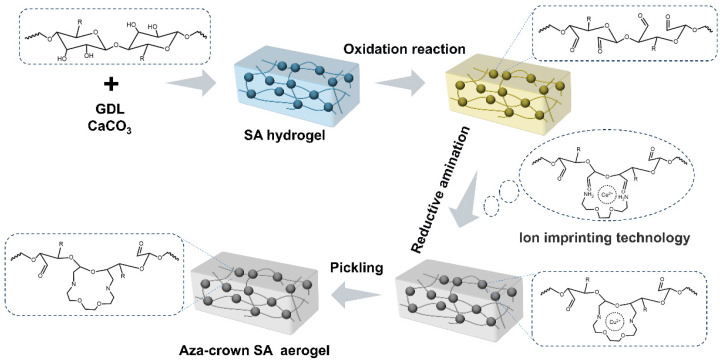
The synthesis mechanism of aza-crown ether functionalized sodium alginate aerogels.

To develop a polysaccharide-based adsorbent with high Cu^2+^ affinity and structural robustness, a molecular-level sugar-ring modification strategy was designed to construct aza-crown ether units in situ within the SA matrix. Unlike conventional surface-grafting or physical blending approaches that merely immobilize crown ethers onto polymer backbones, this strategy chemically generates the aza-crown moieties directly from the saccharide structure, ensuring high Cu^2+^ selectivity, adsorption capacity, and structural stability, and resistance to leaching (as shown in Figure 1).

The synthesis proceeds through a sequential three-step transformation. First, SA hydrogels were formed via a controlled ionic crosslinking process, in which GDL hydrolyzed to gluconic acid, gradually decreasing the pH and triggering the sustained release of Ca^2+^ ions from CaCO_3_. This slow-release mechanism yielded a homogeneous and mechanically stable alginate network [26,27]. Subsequently, periodate oxidation selectively cleaved the vicinal C2–C3 diols of the saccharide units, producing dialdehyde groups while partially opening the sugar rings. The resulting OSA contained reactive carbonyl sites that preserved the polymer’s backbone and served as precursors for subsequent condensation [28,29]. In the next stage, BAEE was introduced as a flexible bifunctional N donor monomer with strong Cu^2+^ coordination capability. Under the Cu^2+^-templated condition, the amino groups of BAEE reversibly condensed with the aldehyde groups of OSA, forming dynamic Schiff-base intermediates that self-organized around the Cu^2+^ centers into macrocyclic arrangements. Cu^2+^ templating directed ring closure and enabled precise cavity formation. Subsequently, the reductive amination stabilized the macrocycles by converting C=N bonds into durable C-N bonds, thereby embedding the aza-crown ether rings covalently within the alginate framework. Finally, the hydrogels were freeze-dried to preserve their hierarchical porosity, yielding aza-crown ether functionalized sodium alginate aerogels featuring macrocycles, abundant N/O coordination sites, and enhanced chemical stability. This in situ ring-closure strategy offers an efficient route to embed molecular recognition functionality into a biopolymer matrix, enabling highly selective adsorption of Cu^2+^.

**Figure 2:**
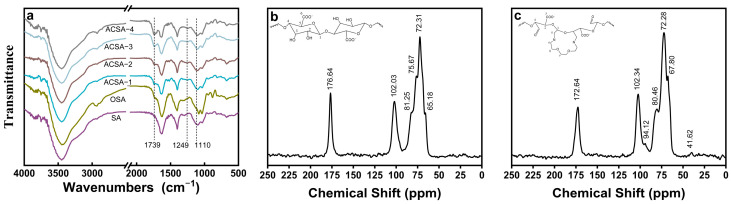
(**a**) FTIR spectra of SA aerogel, OSA and ACSA-1 to ACSA-4; (**b**) ^13^C NMR spectrum of SA aerogel; (**c**) ^13^C NMR spectrum of ACSA.

The chemical and molecular evolution of sodium alginate during oxidation and subsequent aza-crown ether formation were elucidated through Fourier-transform infrared (FTIR) and solid-state ^13^C nuclear magnetic resonance (^13^C NMR) spectroscopy (Figure 2). Oxidation of alginate by sodium periodate introduced a distinct C=O stretching band at 1739 cm^−1^, indicative of aldehyde formation through selective cleavage of the vicinal C2–C3 diols within the pyranose rings [30]. This oxidation partially disrupted the saccharide backbone, generating reactive carbonyl functionalities that served as electrophilic centers for the subsequent condensation reaction. Following the Cu^2+^-templated Schiff-base condensation and reductive amination, the FTIR spectra of ACSA revealed pronounced spectral changes consistent with successful incorporation of aza-crown ether units. The emergence of a strong absorption band at 1110 cm^−1^, corresponding to the C-O-C stretching peak of ether linkages [31], confirmed preservation of the polysaccharide backbone. Meanwhile, a new band at 1249 cm^−1^ was assigned to the C-N stretching vibration of secondary amine groups generated during reductive stabilization [32]. The systematic enhancement of this C-N absorption peak with increasing oxidation degree demonstrates that higher densities of aldehyde groups promote more extensive covalent coupling and intramolecular cyclization, yielding a denser distribution of macrocyclic motifs.

The molecular-level evolution was further confirmed by ^13^C NMR spectra (Figure 2b,c). Pristine SA exhibited characteristic resonances at 65.18, 72.31, 75.67, 81.25, 102.03, and 176.64 ppm, corresponding to carbons in the guluronic (G) and mannuronic (M) units and the carboxyl carbon [33,34]. After functionalization, two additional peaks appeared at 41.62 and 94.12 ppm, assigned to aliphatic carbons adjacent to nitrogen (C-N) and ether-linked carbons (C-O-C), respectively [35,36]. The emergence of these new resonances, together with slight chemical-shift variations in the backbone signals, provides clear evidence of the covalent incorporation of nitrogen and oxygen-containing groups resulting from the Cu^2+^-templated condensation and reductive amination.

**Figure 3:**
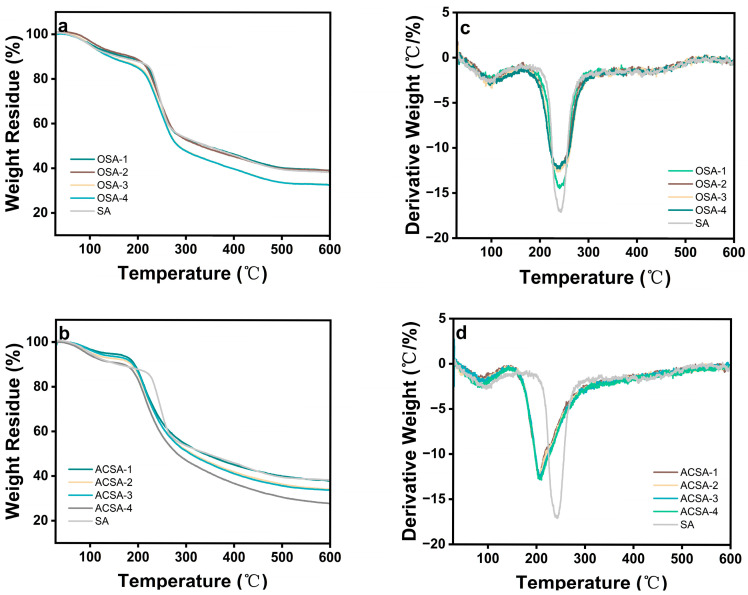
(**a**) TG of SA aerogel and OSA-1 to OSA-4; (**b**) TG of ACSA-1 to ACSA-4; (**c**) DTG of SA aerogel and OSA-1 to OSA-4; (**d**) DTG of ACSA-1 to ACSA-4.

Thermogravimetric analysis (TGA) was conducted to investigate the thermal behavior and structural integrity of pristine sodium alginate, OSA, and ACSA (Figure 3). All samples exhibited two distinct stages of mass loss. The initial weight reduction below 120 °C corresponds to the desorption of physically adsorbed and bound water. The principal degradation stage occurred between 200 °C and 300 °C, associated with the cleavage of hydroxyl and glycosidic bonds in the alginate backbone and the degradation of aldehyde groups introduced during oxidation. The pristine SA aerogel displayed a total mass loss of 61.4% at 600 °C with a maximum decomposition temperature (*T_max_*) of 242 °C, consistent with the typical degradation profile of alginate-based materials. In contrast, OSA samples exhibited slightly higher mass losses (61.5–68.1%) and lower *T_max_* values (235–241 °C), indicating that the cleavage of vicinal diols during periodate oxidation weakened the structural cohesion of the polymer. The progressive decrease in *T_max_* with increasing oxidation degree reflects a trade-off between the introduction of reactive aldehyde groups and the partial loss of backbone rigidity. Further incorporation of aza-crown ether units resulted in a more pronounced decline in thermal stability. ACSA demonstrated earlier onset of degradation and lower *T_max_* values than SA with a trend that intensified with increasing crown ether content. This reduction arose from the formation of flexible C-N and C-O-C linkages within the network, whose bond dissociation energies were inherently lower than those of the native glycosidic bonds. The observed thermal shift also correlated with the relatively low intrinsic decomposition temperature (~200 °C) of the aza-crown ether moiety. The systematic decrease in *T_max_* and the corresponding changes in mass loss behavior provided strong indirect evidence of successful chemical modification. The transition from SA to OSA and ultimately to ACSA illustrated a progressive evolution from a rigid polysaccharide framework to a hybrid network containing covalently anchored N/O-rich macrocyclic motifs.

### 2.2. Physical and Microstructural Properties of ACSA

**Figure 4:**
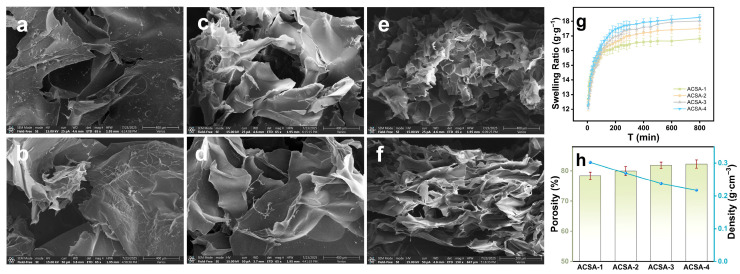
(**a**,**b**) SEM of SA aerogel (65×; 65×); (**c**,**d**) OSA-4 (65×; 65×); (**e**,**f**) ACSA-4 (65×; 150×); (**g**) swelling degree of ACSA-1 to ACSA-4; (**h**) density and porosity values of ACSA-1 to ACSA-4.

The morphological evolution and structural parameters of the aerogels were examined to elucidate how oxidation and aza-crown ether incorporation influence the internal architecture and hydration behavior (Figure 4a–h). The pristine SA aerogel exhibited a dense and relatively smooth lamellar morphology (Figure 4a,b), characteristic of a tightly crosslinked polysaccharide network. After periodate oxidation, the OSA structure became more wrinkled and discontinuous (Figure 4c,d), reflecting the partial cleavage of saccharide rings and disruption of hydrogen-bonding interactions during dialdehyde formation. Upon the Cu^2+^-templated condensation and reductive amination, ACSA (Figure 4e,f) developed a highly porous, interconnected lamellar-wrinkled framework. This transformation indicated the reorganization of crosslinking points and the covalent incorporation of flexible C-N and C-O-C linkages associated with the aza-crown ether units.

The swelling behavior further corroborated the structural tunability of the ACSA series (Figure 4g). All samples reached equilibrium within approximately 600 min, exhibiting swelling ratios between 16.8 and 18.3 g·g^−1^. The low swelling degree confirmed the good dimensional integrity. The slight increase in water uptake with oxidation degree reflected the enhanced porosity and improved diffusion pathways provided by the open 3D network. Quantitatively, the porosity increased from 78.4% for ACSA-1 to 82.3% for ACSA-4, while the bulk density decreased from 0.30 to 0.22 g·cm^−3^ (Figure 4h). These variations confirmed that higher oxidation degrees and increased crown-ether density promoted network expansion and pore development. Such high porosity facilitated rapid water penetration and efficient ion transport, which are critical for achieving fast and selective Cu^2+^ adsorption. Despite the higher porosity, all ACSA maintain excellent dimensional integrity, demonstrating that the structural stability was imparted by covalently integrated aza-crown ether linkages. The combined morphological, porosity, and swelling analyses revealed a progressive structural evolution from a compact alginate network to a flexible yet stable macrocyclic framework. The optimized architecture of ACSA-4, featuring the highest density of aza-crown units as shown in the FTIR, achieved the ideal balance between porosity and structural stability.

### 2.3. Evaluation of Adsorption Properties of ACSA

**Figure 5:**
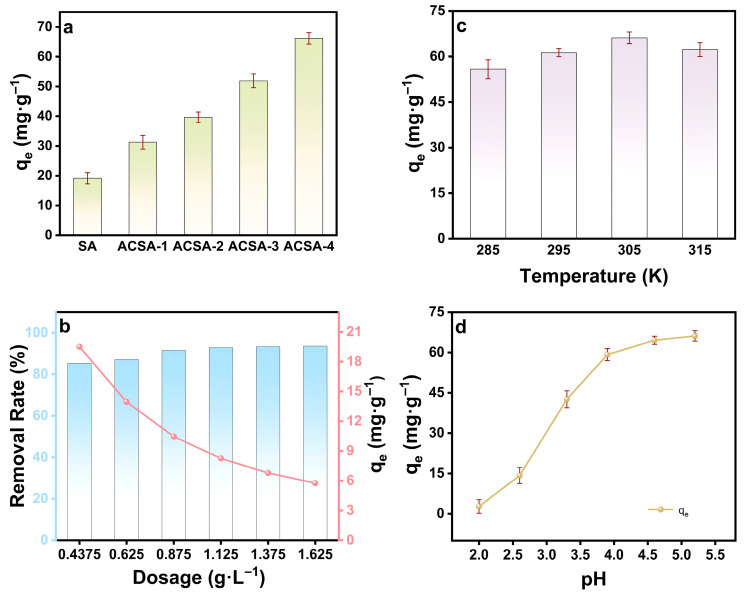
Factors influencing the adsorption capacity of ACSA: (**a**) The effect of OSA oxidation degree; (**b**) effect of the dosage, C = 10 mg·L^−1^; (**c**) effect of the temperature; (**d**) effect of the pH. All parameters were V = 80 mL, m = 50 mg, C = 50 mg·L^−1^, temperature = 305 K and pH 5.2 unless otherwise specified.

The adsorption behavior of the aza-crown ether functionalized sodium alginate aerogels exhibited a clear dependence on molecular configuration and oxidation level. As shown in Figure 5a, the equilibrium adsorption capacity for Cu^2+^ increased from 31.28 mg·g^−1^ (ACSA-1) to 66.15 mg·g^−1^ (ACSA-4), revealing a distinct structure-performance correlation. The progressive enhancement directly reflected the enrichment of N/O coordination domains introduced by the aza-crown ether formation, which effectively increased both the density and accessibility of metal-binding sites within the alginate framework. The superior adsorption performance arose from the cooperative coordination mechanism of the covalently integrated aza-crown ether rings. Each macrocyclic unit provided a preorganized cavity containing nitrogen and oxygen donor atoms positioned at optimal distances for Cu^2+^ chelation. The interaction was stabilized by mixed N/O coordination and the Jahn-Teller distortion, which favored planar geometries and strong ligand-field stabilization. The presence of polar C-N and C-O-C linkages further enhanced dipolar polarization and electron delocalization around the donor atoms, reinforcing the metal-ligand interaction. At the structural level, the hierarchical porosity of ACSA promoted solvent infiltration and ion diffusion, coupling efficient mass transport with molecular-level recognition. This interplay between macrocyclic coordination chemistry and porous topology yielded a synergistic improvement in both adsorption capacity and kinetics. Such dual contributions, electronic and structural, defined the unique performance characteristics of the ACSA series. The systematic increase in Cu^2+^ affinity with higher aza-crown ether density highlighted how electronic coordination geometry and supramolecular architecture could be harmonized within a biopolymeric matrix. The optimized design of ACSA-4 demonstrates that integrating crown-ether-like recognition motifs into renewable polysaccharides provides a chemically stable, highly selective, and sustainable platform for targeted metal-ion capture and water purification.

The adsorption performance of ACSA was strongly governed by adsorbent dosage, which dictates both the number and the utilization efficiency of coordination-active sites. As shown in Figure 5b, at an initial Cu^2+^ concentration of 10 mg·L^−1^, the removal efficiency increased sharply with dosage, whereas the equilibrium adsorption capacity decreased gradually. At low dosages (≤0.4375 g·L^−1^), the scarcity of aza-crown ether sites limited ion uptake. Increasing the dosage to 0.875 g·L^−1^ enabled over 90% removal, reducing the residual Cu^2+^ concentration to 0.848 mg·L^−1^ below the WHO guideline for drinking water [37]. Further dosage increase yielded only marginal efficiency gains (93.6% at 1.625 g·L^−1^), but reduced *q_e_* due to partial underutilization of active sites. This behavior reflects a saturation regime in which the available Cu^2+^ ions become insufficient relative to the number of coordination centers. Thus, the optimal dosage (0.875 g·L^−1^) represents the best compromise between binding-site accessibility, coordination efficiency, and material economy.

The enhanced Cu^2+^ removal at intermediate dosages originates from site-specific chelation within the aza-crown ether cavities, where nitrogen and oxygen donor atoms cooperatively coordinate Cu^2+^ to form stable inner-sphere complexes. Each macrocyclic domain acts as a selective molecular receptor, conferring high binding affinity through geometric and electronic complementarity. The subsequent decline in *q_e_* at higher dosages stems not from reduced chemical affinity but from statistical underutilization of available coordination sites per unit mass.

Temperature-dependent adsorption experiments further elucidate the thermodynamic characteristics of the process (Figure 5c). The equilibrium capacity increased from 55.82 mg·g^−1^ at 285 K to a maximum of 66.15 mg·g^−1^ at 305 K, suggesting that moderate thermal activation enhances diffusion and interfacial mass transfer. However, a slight decrease to 62.26 mg·g^−1^ at 315 K indicates the onset of weak endothermic desorption, implying that excessive heating may disrupt the Cu-ligand coordination equilibria. These results confirm that Cu^2+^ binding to the aza-crown ether sites is both energetically favorable and thermally stable within the 285–315 K range, with optimal performance near ambient temperature.

The influence of solution pH (Figure 5d) further underscores the crucial role of coordination chemistry in dictating the adsorption efficiency. At strongly acidic conditions (pH 2.0), the adsorption capacity was limited to 2.72 mg·g^−1^ due to extensive protonation of the N/O donor atoms, which competitively inhibited Cu^2+^ complexation [38,39]. As the pH increased, proton activity declined, restoring electron-donor availability and facilitating metal coordination within the aza-crown cavities. The adsorption capacity rose progressively to a maximum of 66.15 mg·g^−1^ at pH 5.2, beyond which partial hydrolysis of Cu^2+^ species likely occurs. These findings demonstrate that a mildly acidic environment optimally balances protonation suppression and Cu^2+^ speciation, enabling efficient chelation via synergistic N/O coordination while preserving the structural integrity of the adsorbent. These results indicate that Cu^2+^ adsorption on ACSA is dominated by chemically driven, site-specific coordination and is further modulated by diffusion and protonation equilibria. The robust performance across practical conditions underscores the intrinsic stability and tunable affinity of the aza-crown ether functionalized sodium alginate framework.

### 2.4. Analysis of Adsorption Mechanisms

**Figure 6:**
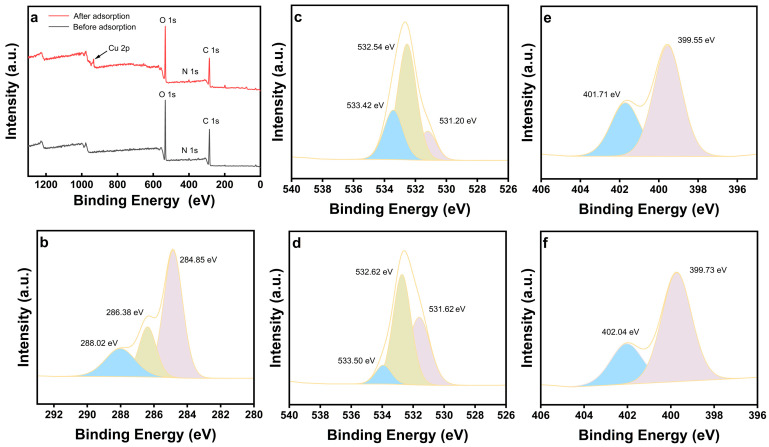
XPS Spectra of ACSA: (**a**) Survey spectra before and after adsorption for Cu^2+^; (**b**) C 1s spectrum before adsorption; (**c**,**d**) O 1s spectra before and after adsorption; (**e**,**f**) N 1s spectra before and after adsorption.

To elucidate the binding mechanism, XPS was performed on ACSA before and after Cu^2+^ adsorption (Figure 6). Prior to adsorption, the survey spectrum exhibited characteristic C 1s, O 1s, and N 1s peaks, confirming the expected composition of the aza-crown ether functionalized sodium alginate matrix. The high-resolution C 1s spectrum (Figure 6b) showed three components at 284.85, 286.38, and 288.02 eV, corresponding to aliphatic carbon (C-C/C-H) from the alginate backbone [40], C-O/C-N species (C-OH, C-O-C, C-N) originating from the aza-crown ether structure [41,42], and carboxyl carbon (O-C=O) groups [43]. The O 1s spectrum (Figure 6c) contained peaks at 531.20, 532.54, and 533.42 eV, assigned, respectively, to oxygen in carboxyl, ether, and hydroxyl environments [44,45,46,47]. Meanwhile, the N 1s spectrum (Figure 6e) presented two distinct signals at 399.55 eV (-NH-) and 401.71 eV (protonated -NH_2_^+^) [48,49], confirming the successful incorporation of nitrogen-bearing functional sites. After Cu^2+^ adsorption, the survey spectrum (Figure 6a) revealed the emergence of a clear Cu 2p doublet centered at 934.29 eV, confirming the immobilization of copper species within the aerogel framework [50]. In the O 1s region (Figure 6d), the ether-related C-O-C component shifted from 532.54 eV to 532.62 eV, indicating coordination between Cu^2+^ and ether oxygen atoms. Concurrently, in the N 1s spectrum (Figure 6f), both -NH- and -NH_2_^+^ peaks shifted toward higher binding energies (from 399.55/401.71 eV to 399.73/402.04 eV), reflecting electron donation from nitrogen atoms to the Cu^2+^ center. The simultaneous positive shifts in O 1s and N 1s binding energies signified a reduction in local electron density around the donor atoms, consistent with ligand-to-metal charge transfer during complexation.

**Figure 7:**
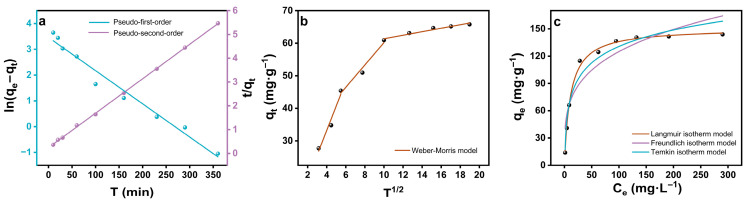
Model fits for adsorption kinetics and isotherms of ACSA: (**a**) pseudo-first-order and pseudo-second-order kinetic models; (**b**) Weber-Morris intraparticle diffusion model; (**c**) Langmuir, Freundlich, and Temkin isotherm models (C_0_ = 10–380 mg·L^−1^). Unless otherwise specified: V = 80 mL, m = 50 mg, C = 50 mg·L^−1^, temperature = 305 K and pH 5.2.

To elucidate the kinetics and governing mechanism of Cu^2+^ uptake, the experimental data were analyzed using the pseudo-first-order (Equation (5)), pseudo-second-order (Equation (6)), and Weber-Morris intraparticle diffusion (Equation (7)) models [51,52], and the corresponding fitting parameters are summarized in Table 1. As shown in Figure 7, the adsorption capacity (*q_t_*) increased sharply during the first 60 min, reaching approximately 50 mg·g^−1^, and gradually approached equilibrium after 120 min. Among the three models, the pseudo-second-order kinetic model exhibited the best correlation (R^2^ = 0.999), and the calculated equilibrium capacity (*q_e,cal_* = 69.11 mg·g^−1^) closely matched the experimental value (*q_e,exp_* = 66.15 mg·g^−1^). This strong correlation indicated that the adsorption process was predominantly controlled by chemisorption involving valence sharing or electron exchange between Cu^2+^ ions and the active coordination sites. Such behavior aligned with the N/O-bidentate binding mechanism evidenced by XPS analysis, confirming that chemical complexation rather than mere physical adsorption dominated Cu^2+^ capture within the aza-crown ether domain.

The intraparticle diffusion model provides further insight into the adsorption dynamics. The *q_t_* versus *t^1/2^* plot displayed three linear segments, corresponding to (i) boundary-layer diffusion during the initial phase, (ii) gradual intraparticle diffusion through the mesoporous network, and (iii) the final equilibrium stage where diffusion resistance increased as the active sites became saturated. The intercept of each linear region was nonzero (C ≠ 0), signifying that intraparticle diffusion did not act as the sole rate-determining step. Instead, the overall process was jointly governed by surface chemisorption through N/O coordination and partial diffusion within the hierarchically porous framework.

The equilibrium adsorption behavior of Cu^2+^ on ACSA was investigated using the Langmuir, Freundlich, and Temkin isotherm models [53,54,55,56], as described by Equations (8)–(10). The corresponding fitting parameters are summarized in Table 2. As shown in Figure 7c, the Langmuir model provided an excellent fit to the experimental data (R^2^ = 0.996), demonstrating that Cu^2+^ adsorption occurred predominantly through monolayer coverage on a homogeneous distribution of active sites. The calculated maximum adsorption capacity (*q_m_*) reached 150.82 mg·g^−1^, placing ACSA among the most efficient polysaccharide-derived adsorbents reported to date (Table 3). This exceptionally high capacity reflected the strong affinity of the aza-crown ether cavities, where well-defined macrocyclic coordination environments and cooperative N/O donor atoms enabled specific complexation with Cu^2+^ ions.

The Freundlich model exhibited lower correlation, confirming that surface heterogeneity and multilayer formation played a minor role in the overall adsorption process. Meanwhile, the Temkin model indicated a gradual decrease in adsorption enthalpy with increasing surface coverage, signifying moderate adsorbent-adsorbate interactions. The positive Temkin binding constant further revealed that Cu^2+^ adsorption on ACSA was thermodynamically favorable [57]. These findings confirmed that Cu^2+^ removal with ACSA was governed primarily by chemisorptive complexation, complemented by minor contributions from physisorption. The uniform distribution of aza-crown ether rings ensured a high density of energetically equivalent binding domains, where Cu^2+^ ions were captured through strong coordination with nitrogen and oxygen donor atoms in the macrocyclic framework. The hierarchical porosity of the aerogel further facilitated diffusion and accessibility of these active sites, leading to both rapid uptake and high equilibrium capacity.

**Table 3:** Comparison with the maximum adsorption capacity of other adsorbent materials.

Adsorbent	Conditions	*q_m_* (mg·g^−1^)	References
PI aerogel	/	83.3	[58]
GO/CMC	pH = 5.0, T = 30 °C	146.4	[59]
CSFP	pH = 5.0, T = 60 °C	58.26	[60]
GCDiA-4	pH = 3.0, T = 25 °C	149.62	[61]
PGA-AP	pH = 6.0, T = 60 °C	78.99	[62]
CSVT	pH = 8.0, T = 50 °C	116.22	[63]
PNIPAM NG	pH = 6.5–7.0, T = 30 °C	91.12	[64]
RM-CIIP-3	pH = 5.5, T = 25 °C	94.64	[65]
ACSA-4	pH = 5.2, T = 32 °C	150.82	This Work

Through XPS binding energy shifts, swelling and porosity measurements, pronounced pH dependence, and kinetic and isotherm fitting model analysis, it was determined that during the adsorption of copper ions by ACSA, the matrix primarily provides a hydrophilic, ion-accessible porous carrier, while N/O sites within the engineered aza-crown cavity serve as the key source for Cu^2+^ adsorption.

### 2.5. Selective Adsorption Performance

To evaluate the practical applicability of ACSA in complex ionic environments, competitive adsorption experiments were conducted to assess its selectivity for Cu^2+^ in the presence of coexisting divalent cations typically found in wastewater. Tests were performed at an initial total metal concentration of 200 mg·L^−1^ under both binary (Cu^2+^/Zn^2+^, Cu^2+^/Cd^2+^, Cu^2+^/Ni^2+^) and quaternary (Cu^2+^/Zn^2+^/Cd^2+^/Ni^2+^) systems. NIACSA served as the control group for the same adsorption experiment.

**Figure 8:**
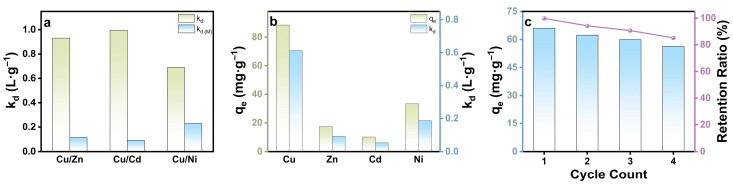
(**a**) Co-existing system of Cu^2+^ and a single metal-ion; (**b**) Co-existing system of Cu^2+^ and multiple metal-ions; (**c**) The recyclability of ACSA-4.

As shown in Figure 8, ACSA consistently exhibited strong Cu^2+^ preference across all systems, maintaining high adsorption efficiency even under multicomponent competitive conditions. In binary mixtures, the distribution coefficients (*k_d_*) for Cu^2+^ were 0.93, 0.99, and 0.68 L·g^−1^ for the Cu/Zn, Cu/Cd, and Cu/Ni pairs, respectively, substantially higher than those of the competing ions Zn^2+^ (0.11 L·g^−1^), Cd^2+^ (0.09 L·g^−1^), and Ni^2+^ (0.23 L·g^−1^). Even in the more competitive quaternary system, ACSA retained a high Cu^2+^ adsorption capacity of 88.47 mg·g^−1^ and a *k_d_* of 0.61 L·g^−1^, confirming its superior ion-recognition capability in multi-ion environments. For NIACSA, in binary mixed systems, the distribution coefficients of Cu^2+^ in Cu/Zn, Cu/Cd, and Cu/Ni systems are very close to those of competing ions, failing to demonstrate selective adsorption capacity. In quaternary competitive adsorption systems, NIACSA exhibited distribution coefficients of 0.27, 0.18, 0.15, and 0.23 L·g^−1^ for Cu^2+^, Zn^2+^, Cd^2+^, and Ni^2+^, respectively, similarly failing to demonstrate selective adsorption capacity. Compared to NIACSA, ACSA exhibits significant selective recognition capabilities in both binary and multi-component competitive ion systems (Figure S4). Additionally, the adsorption capacities of ACSA-4 for Cu^2+^ in CuCl_2_·2H_2_O and Cu(NO_3_)_2_·5H_2_O solutions were systematically compared to assess anion effects. The results show that Cl^−^ and NO_3_^−^ exert negligible influence on *q_e_*, giving rise to comparable adsorption capacities (Table S2). Consequently, under conditions involving common background anions NO_3_^−^ and low to moderate Cl^−^ concentrations, the observed selectivity is dominated by cavity-defined N/O coordination rather than anion-dependent effects.

The template enhances “cavity-defined coordination selectivity” rather than generalized N/O surface adsorption. The outstanding selectivity of ACSA for Cu^2+^ arises from its well-matched coordination geometry and cavity size. The ionic radius of Cu^2+^ (0.73 Å) closely aligns with the internal diameter of 15-crown-5 (≈1.7–2.2 Å), enabling an optimal fit and the formation of highly stable host-guest complexes. Moreover, due to the Jahn-Teller effect [66], Cu^2+^ readily undergoes distortion that stabilizes its coordination environment within the macrocyclic cavity, further enhancing binding affinity. Although Zn^2+^ (0.74 Å) has a similar ionic radius, its harder-acid nature, as described by HSAB theory, favors coordination with oxygen rather than nitrogen donors [67,68], leading to weaker interactions in the mixed N/O-donor environment of the aza-crown ether. Cd^2+^, with its substantially larger radius (0.95 Å) [69], is sterically hindered from entering the crown-ether cavity, resulting in poor complexation. In contrast, Ni^2+^ (0.69 Å) is smaller than Cu^2+^, but forms less stable complexes and lacks the additional stabilization associated with the Jahn-Teller effect, giving rise to inferior competitive adsorption [70]. In addition, the hydroxyl-rich sodium alginate matrix contributes through hydrogen bonding, optimizing the spatial configuration and accessibility of the imprinted cavities. These results highlight that the selective adsorption behavior of ACSA originates from the synergistic integration of molecular recognition and structural optimization. The aza-crown ether rings offer highly specific coordination domains with N/O bidentate donor functionality, while the hierarchical porosity of the framework ensures rapid ion diffusion and full site accessibility. This combination enables ACSA to achieve both high selectivity and substantial adsorption capacity, even in multicomponent systems. The ability of ACSA to maintain selective and stable Cu^2+^ capture in the presence of competing ions underscores its potential as a robust, sustainable, and scalable material for targeted copper recovery and advanced wastewater treatment applications.

### 2.6. Regeneration Performance and Reusability

Reusability is a critical parameter in evaluating the practical viability and environmental sustainability of adsorbent materials. The regeneration performance of ACSA was assessed through consecutive Cu^2+^ adsorption–desorption cycles using a mild acidic eluent to remove the captured ions. The desorption process proceeded efficiently, and the aerogel maintained its structural and functional integrity owing to the robust three-dimensional framework formed by Ca^2+^ crosslinking of the alginate carboxyl groups. As shown in Figure 8c, ACSA exhibited excellent durability, retaining over 80% of its initial adsorption capacity after four successive cycles, measurements revealed only minimal mass loss (Figure S2). The minor mass loss resulted in the partial loss of active groups, leading to a slight decrease in adsorption capacity. At the same time, this also indicates that the aza-crown ether coordination sites and the alginate network remained chemically stable during regeneration, with negligible leaching or degradation, which was verified by FTIR (Figure S3). The preservation of adsorption efficiency also confirmed the reversibility of the Cu^2+^ binding process, in which proton-assisted ligand exchange effectively regenerated the active N/O donor sites. Beyond its chemical robustness, the biopolymeric nature of the sodium alginate matrix offers a distinct environmental advantage. The material is biodegradable, nontoxic, and easily disposable, minimizing the risk of secondary contamination after prolonged application. Taken together, the high regeneration efficiency, structural resilience, and intrinsic biodegradability highlight ACSA as a sustainable and economically viable adsorbent capable of long-term operation in continuous or batch wastewater treatment systems. This combination of performance and environmental compatibility establishes ACSA as a promising platform for selective Cu^2+^ recovery and circular-resource applications.

## 3. Conclusions

This work introduces a sustainable molecular-engineering strategy for constructing aza-crown ether functionalized sodium alginate aerogels that unite precise molecular recognition with a biodegradable and structurally robust biopolymer matrix. Through sequential saccharide-ring oxidation, Cu^2+^-templated self-assembly, and reductive amination, aza-crown ether units were covalently integrated into the alginate framework, yielding a hierarchically porous, mechanically stable network with macrocyclic binding sites. Comprehensive characterization verified the successful in situ construction of the aza-crown architecture and elucidated its structure-property correlations. The resulting aerogels, ACSA, displayed exceptional adsorption performance and stability. Kinetic analysis confirmed pseudo-second-order behavior (R^2^ = 0.999) with a Langmuir maximum capacity of 150.82 mg·g^−1^, evidencing a chemisorption-dominated process governed by N/O coordination within the macrocyclic cavities. ACSA maintained efficient Cu^2+^ uptake across broad pH, temperature, and dosage ranges, and exhibited pronounced selectivity over Zn^2+^, Cd^2+^, and Ni^2+^. This outstanding selectivity stems from the synergistic alignment of cavity size and coordination geometry, where the Cu^2+^ ionic radius (0.73 Å) and Jahn-Teller distortion enable optimal fit and enhanced stability within the aza-crown cavity, while mismatched radii and electronic configurations of competing cations lead to weaker complexation. The alginate matrix further contributes through hydrogen bonding and pore accessibility, supporting both molecular recognition and mass transport. Even after four regeneration cycles, ACSA retained more than 80% of its initial capacity, demonstrating superior regenerability, structural resilience, and environmental safety.

Beyond its performance metrics, this saccharide-ring modification approach resolves the intrinsic limitations of conventional crown-ether immobilization such as leaching, weak anchoring, and limited functional density by chemically integrating the macrocyclic units into a renewable polysaccharide framework. This molecularly precise, environmentally benign design establishes a scalable strategy for developing next-generation bio-based adsorbents capable of targeted metal-ion recognition and recovery. The concept demonstrated here extends beyond copper purification, offering a versatile platform for engineering selective coordination materials for environmental remediation, catalysis, and resource circularity.

## 4. Materials and Methods

### 4.1. Materials

Sodium alginate (SA, CP grade, viscosity = 200 ± 10 mPa·s), calcium carbonate (AR), sodium periodate (AR), tert-butanol (AR), 1,2-bis(2-aminoethoxy)ethane (BAEE, 98%), sodium triacetoxyborohydride (NaBH(OAc)_3_, AR), cupric acetate anhydrous (AR), ethylene glycol (GC), acetic acid (AR), zinc chloride (AR), copper(II) chloride dihydrate (AR), nickel(II) chloride hexahydrate (AR), and potassium bromide (AR) were purchased from Macklin Reagent Co. (Shanghai, China). D-(+)-Gluconic acid δ-lactone (GDL, AR grade), Copper(II) nitrate pentahydrate (AR) and cadmium chloride monohydrate (AR) were obtained from Aladdin Reagent Co. (Shanghai, China). Dichloromethane (DCM), dimethyl sulfoxide (DMSO), and hydrochloric acid (HCl) were purchased from Xilong Chemical Co. (Guangzhou, China). All chemicals were of analytical grade and used as received without further purification. Deionized water was freshly prepared in the laboratory and used for all experiments.

### 4.2. Preparation of Oxidized Sodium Alginate Aerogel

SA hydrogels were first prepared via an internal gelation route. In a typical procedure, 1.0 g of SA powder was dissolved in 25 mL of deionized water under magnetic stirring at room temperature until a transparent solution was obtained. Subsequently, 0.10 g of calcium carbonate (CaCO_3_) was added and uniformly dispersed, followed by the dropwise addition of 0.34 g of GDL under vigorous stirring. The mixture was immediately poured into molds and allowed to stand at ambient temperature for 4 h to complete gelation. The resulting hydrogels were cut into rectangular blocks (1.5 × 1.0 × 0.2 cm^3^) for subsequent oxidation. Oxidation was performed by immersing the hydrogels in aqueous sodium periodate (NaIO_4_) solutions containing 0.5, 0.7, 0.9, or 1.1 g of NaIO_4_ dissolved in 125 mL of deionized water. The reaction proceeded at 32 °C in the dark for 10 h to prevent photodecomposition of periodate ions. After oxidation, 3 mL of ethylene glycol was added to terminate the reaction, and the samples were thoroughly rinsed with deionized water to remove unreacted species. To minimize capillary-induced shrinkage during drying, the oxidized hydrogels were solvent-exchanged with tert-butanol and then freeze-dried to yield oxidized sodium alginate aerogels (OSA). The aerogels obtained at different oxidation degrees were designated as OSA-1, OSA-2, OSA-3, and OSA-4, respectively. The corresponding oxidation levels were determined by aldehyde titration as described in the Supplementary Materials.

### 4.3. Preparation of Aza-Crown Ether Functionalized Alginate Aerogel

The incorporation of aza-crown ether (ACE) units into oxidized sodium alginate aerogels was achieved through a Cu^2+^-templated reductive amination strategy. In a typical procedure, the pre-oxidized OSAs were dispersed in 60 mL of dichloromethane containing a catalytic amount of acetic acid under a nitrogen atmosphere to ensure anhydrous conditions. Cupric acetate anhydrous [Cu(OAc)_2_, 1.0 equiv per aldehyde group], dissolved in 2 mL of dimethyl sulfoxide (DMSO), was introduced as the Cu^2+^ template to guide the macrocyclic coordination geometry. Subsequently, 1,2-bis(2-aminoethoxy) ethane (BAEE, 4.1 equiv relative to the aldehyde groups) was added to initiate condensation with the aldehyde functionalities on the alginate chains. Sodium triacetoxyborohydride [NaBH(OAc)_3_] was then added dropwise as a mild reducing agent to promote in situ reductive amination and stabilize the newly formed aza-crown ether moieties. The reaction mixture was magnetically stirred for 5 h at room temperature, enabling uniform Cu^2+^-templated formation of ACE cavities throughout the aerogel framework.

Upon completion, the reaction was quenched with dilute hydrochloric acid, and the solid product was collected and purified by sequential washing with 0.1 M HCl and deionized water until the filtrate reached neutral pH. The purified samples were freeze-dried to obtain aza-crown ether functionalized sodium alginate aerogels (ACSA). The materials synthesized from OSA-1, OSA-2, OSA-3, and OSA-4 precursors were designated as ACSA-1, ACSA-2, ACSA-3, and ACSA-4, respectively. Additionally, following the aforementioned steps, an aerogel without added template copper ions was prepared and named NIACSA.

### 4.4. Characterization

The chemical structure, coordination environment, and morphological features of the samples were comprehensively characterized using complementary analytical techniques. Fourier-transform infrared spectroscopy (FTIR, Bruker NIOS, Bielefeld, Germany) was carried out over the 500–4000 cm^−1^ range (KBr pellet method) to monitor functional group evolution during the transformation of SA into OSA and ACSA. The elemental composition and electronic states of surface species were analyzed by X-ray photoelectron spectroscopy (XPS, Thermo Fisher Scientific Escalab 250Xi, Waltham, MA, USA), and all spectra were deconvoluted using Avantage software (version 6.8.1) to identify coordination interactions between Cu^2+^ and aza-crown ether moieties. Solid-state ^13^C nuclear magnetic resonance (NMR, Bruker AVANCE II 600 MHz, Bielefeld, Germany) spectroscopy was used to probe the local carbon environments and verify the successful incorporation of aza-crown ether units into the alginate framework. Thermogravimetric analysis (TGA, JingYiGaoKe ZCT1, Beijing, China) was performed under a nitrogen atmosphere from room temperature to 600 °C at a heating rate of 10 °C·min^−1^ to assess the thermal stability and compositional integrity of the materials. The surface morphology and porous architecture of gold-sputtered samples were visualized by field-emission scanning electron microscopy (FE-SEM, Verios G4 UC, Waltham, MA, USA).

### 4.5. Swelling and Porosity of ACSA

The swelling behavior of ACSA was examined to evaluate their water uptake capacity and network stability. Rectangular ACSA aerogel samples (1.5 cm × 1.0 cm × 0.2 cm; initial dry mass = *M_a_*) were immersed in 100 mL of deionized water at room temperature. At specified time intervals, the samples were removed, gently blotted with filter paper to eliminate surface moisture, and immediately weighed (*M_b_*). Each measurement was performed in triplicate, and the swelling ratio (*S*) (g·g^−1^) was determined using Equation (1).

(1) $$S=\frac{M_{a}-M_{b}}{M_{a}}$$

The density and porosity of ACSA were evaluated to characterize their structural compactness and internal pore architecture. The apparent density (*ρ_a_*) was determined from the ratio of the aerogel’s dry mass to its geometric volume, measured prior to compression. The skeletal density (*ρ_b_*) was obtained after compaction by measuring the mass and volume of the same samples under identical conditions. All measurements were performed in triplicate to ensure accuracy and reproducibility. The total porosity (*P*) was then calculated using Equation (2):

(2) $$P(\%)=(1-\frac{\rho_{a}}{\rho_{b}})\times 100\%$$

### 4.6. Adsorption Experiment of ACSA

Batch adsorption tests were performed to evaluate the Cu^2+^ removal efficiency of ACSA and examine the factors influencing their sorption behavior. Pre-weighed aerogel samples were immersed in 80 mL of Cu^2+^ solution of known initial concentration. The suspensions were agitated at 100 rpm using a thermostatic orbital shaker (SHA-CA, Jingbo, Changzhou, China) to ensure uniform mixing and effective mass transfer between the solid and liquid phases. At specified time intervals, aliquots of the supernatant were collected, filtered to remove any suspended particles, and analyzed for Cu^2+^ concentration using atomic absorption spectrometry (AAS, Agilent 240 DUO, Santa Clara, CA, USA).

To elucidate the factors influencing adsorption, systematic studies were conducted under varying conditions, including the oxidation degree of OSA, adsorbent dosage, initial Cu^2+^ concentration, and solution pH. All experiments were conducted in triplicate to ensure data reproducibility. The equilibrium adsorption capacity (*q_e_*, mg·g^−1^) was calculated according to Equation (3):

(3) $$q_{e}=\frac{\left(C_{0}-C_{e}\right)}{m}\times V$$

where *C_0_* and *C_e_* (mg·L^−1^) denote the initial and equilibrium Cu^2+^ concentrations, *V* (L) is the solution volume, and *m* (g) represents the dry mass of the adsorbent.

### 4.7. Selectivity of ACSA

The competitive adsorption behavior of ACSA toward Cu^2+^ was examined in the presence of other divalent cations (Zn^2+^, Cd^2+^, and Ni^2+^). Binary systems (Cu^2+^/Zn^2+^, Cu^2+^/Cd^2+^, and Cu^2+^/Ni^2+^) and a quaternary mixture containing all four ions were prepared, with each metal ion adjusted to an initial concentration of 200 mg·L^−1^. For each test, 50 mg of ACSA was added to 80 mL of the prepared solution and agitated at 100 rpm in a thermostatic shaker at 305 K to ensure uniform dispersion and sufficient contact between the adsorbent and metal ions. The suspensions were agitated until equilibrium was reached, ensuring consistent mass-transfer conditions across all samples. After equilibration, the supernatants were collected and analyzed for residual ion concentrations using inductively coupled plasma atomic emission spectrometry (ICP-AES, Agilent 5110, Santa Clara, CA, USA). The distribution coefficient (*k_d_*, L·g^−1^) for each metal ion was determined according to Equation (4):

(4) $$k_{d}=\frac{q_{e}}{C_{e}}$$

where *q_e_* (mg·g^−1^) represents the equilibrium adsorption capacity and *C_e_* (mg·L^−1^) denotes the equilibrium concentration of the ion in solution.

### 4.8. Reusability

The regeneration performance and reusability of ACSA were evaluated through four consecutive adsorption–desorption cycles to assess their long-term stability. After each adsorption experiment, the used aerogels were desorbed using 0.1 M HCl as the eluent to remove the adsorbed Cu^2+^ ions. The samples were then thoroughly rinsed with deionized water until a neutral pH was achieved, ensuring complete elimination of residual acid. The regenerated aerogels were subsequently freeze-dried before being reused in the next adsorption cycle under identical experimental conditions. The adsorption capacity obtained after each cycle was recorded and compared to the initial value to quantify the regeneration efficiency and evaluate the structural integrity and durability of ACSA.

### 4.9. Methodologies for Adsorption Mechanism

#### 4.9.1. Adsorption Kinetics of ACSA for Cu^2+^

The adsorption kinetics of Cu^2+^ onto ACSA were investigated to elucidate the rate-controlling mechanisms and adsorption dynamics. In each test, 50 mg of ACSA was added to 80 mL of Cu^2+^ solution with an initial concentration of 50 mg·L^−1^. The mixtures were agitated in a thermostatic shaker at 305 K, and aliquots of the supernatant were withdrawn at predetermined time intervals to measure the residual Cu^2+^ concentration (*C_t_*) using atomic absorption spectrometry (AAS, Agilent 240 DUO, Santa Clara, CA, USA).

The adsorption capacity at time t (*q_t_*, mg·g^−1^) was determined from Equation (3), substituting *C_t_* for the equilibrium concentration (*C_e_*). The kinetic data were analyzed using three classical models, the pseudo-first-order, the pseudo-second-order, and the Weber-Morris intraparticle diffusion, represented by Equations (5)–(7):

(5) $$\ln(q_{e}-q_{t})=\ln q_{e}-k_{1}t$$

(6) $$\frac{t}{q_{t}}=\frac{1}{k_{2}{q_{e}}^{2}}+\frac{t}{q_{e}}$$

(7) $$q_{t}=k_{w}\times t^{\frac{1}{2}}+C$$

where *q_t_* (mg·g^−1^) is the adsorption capacity at time t; *q_e_* (mg·g^−1^) is the equilibrium adsorption capacity; *k_1_* (min^−1^) and *k_2_* (g·mg^−1^·min^−1^) are the pseudo-first-order and pseudo-second-order rate constants, respectively; and *k_w_* (mg·g^−1^·min^−0.5^) and C represent the intraparticle diffusion rate constant and the boundary layer thickness.

#### 4.9.2. Adsorption Isotherm of ACSA for Cu^2+^

Adsorption isotherm analysis was performed to characterize the equilibrium interaction between Cu^2+^ and the active sites within the ACSA aerogel framework. The experiments were conducted at 305 K using Cu^2+^ solutions with initial concentrations ranging from 10 to 380 mg·L^−1^, while maintaining constant pH, agitation speed, and adsorbent dosage. After equilibrium was attained, the residual Cu^2+^ concentrations in the filtrates were determined using atomic absorption spectrometry (AAS, Agilent 240 DUO, Santa Clara, CA, USA).

To describe the adsorption mechanism and evaluate the surface characteristics of ACSA, the equilibrium data were fitted to the Langmuir, Freundlich, and Temkin isotherm models. The linearized forms of these models are given as follows:

(8) $$q_{e}=\frac{k_{l}q_{m}C_{e}}{1+k_{l}C_{e}}$$

(9) $$q_{e}=k_{f}C_{e}^{\frac{1}{n}}$$

(10) $$q_{e}=\frac{RT}{b}\ln(AC_{e})$$

where *C_e_* (mg·L^−1^) and *q_e_* (mg·g^−1^) are the equilibrium concentration of Cu^2+^ in solution and the amount adsorbed at equilibrium, respectively. The Langmuir model assumes monolayer adsorption onto a homogeneous surface, where *q_m_* (mg·g^−1^) represents the maximum adsorption capacity and *k_l_* (L·mg^−1^) denotes the affinity constant. The Freundlich model accounts for multilayer adsorption on heterogeneous surfaces, with *k_f_* (mg·L^−1^) as the adsorption capacity constant and 1/n as the heterogeneity factor. The Temkin model considers adsorbent-adsorbate interactions, where *A* (L·g^−1^) is the equilibrium binding constant and *b* (J·mol^−1^) represents the adsorption heat constant.

## Figures and Tables

**Table 1:** Kinetic parameters of the adsorption process of ACSA for Cu^2+^.

Adsorbent	*q_e, exp_* (mg·g^−1^)	Pseudo-First-Order Kinetic Model			Pseudo-Second-Order Kinetic Model				
		*q_e, cal_* (mg·g^−1^)	k_1_ × 10^−2^ (min^−1^)	R^2^	*q_e, cal_* (mg·g^−1^)	k_2_ × 10^−4^ (g·mg^−1^·min^−1^)			R^2^
ACSA	66.15	31.78	1.28	0.972	69.11	8.61			0.999
Adsorbent	Weber-Morris intraparticle diffusion model								
	*k_w1_*	*C_1_*	R^2^	*k_w2_*	*C_2_*	R^2^	*k_w3_*	*C_3_*	R^2^
ACSA	7.52	3.08	0.92	3.43	25.85	0.94	0.06	56	0.93

**Table 2:** Isothermal adsorption parameters of the adsorption process of ACSA for Cu^2+^.

Adsorbent	Langmuir Isotherm Model			Freundlich Isotherm Model			Temkin Isotherm Model		
	*k_l_*	*q_m_*	R^2^	*k_f_*	1/n	R^2^	b	A	R^2^
ACSA	0.091	150.82	0.996	38.26	0.257	0.875	97.35	1.51	0.962

## Data Availability

The original contributions presented in this study are included in the article and Supplementary Materials. Further enquiries can be directed to the corresponding author.

## Supplementary Materials

The following supporting information can be downloaded at: https://www.mdpi.com/article/10.3390/gels12010078/s1, Figure S1: Photographs showing the color change of ACSA before and after Cu^2+^ adsorption, illustrating the strong coordination and complexation of Cu^2+^ ions with the aza-crown ether sites; Table S1: The oxidation degree of OSA. Figure S2. Mass loss of ACSA-4 before and after four regeneration cycles. Figure S3. FTIR spectra of ACSA-4 before and after four regeneration cycles. Figure S4. (a) Coexistence system of Cu^2+^ with a single metal ion using NIACSA; (b) Coexistence system of Cu^2+^ with multiple metal-ions using NIACSA. Table S2. Effect of salt type on Cu^2+^ adsorption capacity. Reference [71] is cited in the Supplementary Materials.
