# Supplementary material for: Molecularly Engineered Aza-Crown Ether Functionalized Sodium Alginate Aerogels for Highly Selective and Sustainable Cu2+ Removal
Source: Gels. 2026 Jan 16;12(1):78. doi: 10.3390/gels12010078 (PMC12841500; doi:10.3390/gels12010078)
Supplement: Supplementary file 1 [file gels-12-00078-s001.zip › gels-4047705-supplementary.pdf]

Supplementary Material

Molecularly engineered aza-crown ether functionalized sodium alginate aerogels for highly selective and sustainable Cu^{2+} removal

Teng Long[1,2,3], Ayoub El Idrissi[1,2,3], Lin Fu[1,2,3], Yufan Liu[1,2,3], Banlian Ruan[1,2,3], Minghong Ma[1,2,3], Zhongxun Li[1,2,3], Lingbin Lu*[,1,2,3]

[1] School of Materials Science and Engineering, Hainan University, Haikou 570228, PR China

[2] State Key Laboratory of Tropic Ocean Engineering Materials and Materials Evaluation, Haikou 570228, PR China

[3] Special Glass Key Lab of Hainan Province, Haikou 570228, PR China

1. Determination of the oxidation degree of OSA

To determine the oxidation degree of oxidized sodium alginate (OSA), varying amounts of sodium periodate (see Table S1) were dissolved in 125 mL of deionized water. Subsequently, the previously prepared 4 wt% sodium alginate (SA) hydrogel was added to the solution and oxidized under continuous stirring at room temperature in the dark. The molar mass of the uronic acid unit in sodium alginate was taken as 198 g·mol^{-1} for calculation purposes. Before quenching the reaction with ethylene glycol, the residual periodate ions (IO_4^-) in the solution were quantified using UV-visible spectrophotometry at λ = 290 nm[1]. A calibration curve was obtained by measuring the absorbance of a series of sodium periodate standard solutions (10-40 mmol·L^{-1}) at

the same wavelength. The oxidation degree (DO) of OSA was then calculated using the following equation：

$$DO = \frac{n_{0-} n_t}{n_s} \times 100\%$$

where n_0 and n_t represent the initial and residual concentrations of sodium periodate, respectively, and n_s denotes the number of moles of uronic acid units in sodium alginate.

Table S1 The oxidation degree of OSA

m_{NaIO4} /g	Mole of Uronic Acid /mmol	Absorbance	DO
0.5	5.05	0.357	5.52%
0.7	5.05	0.496	8.17%
0.9	5.05	0.619	12.68%
1.1	5.05	0.749	16.36%

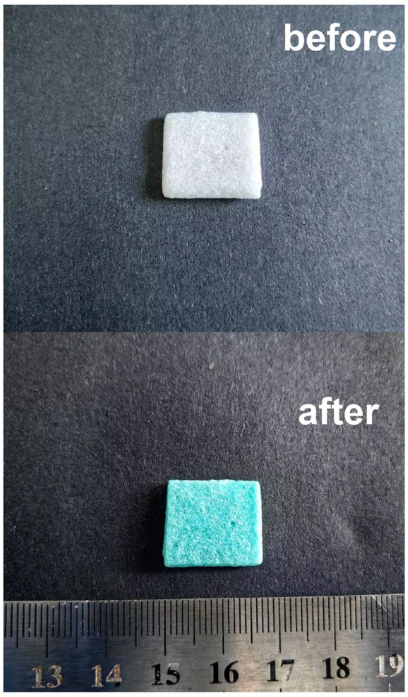

Figure S1. Photographs showing the color change of ACSA before and after Cu^{2+}

adsorption, illustrating the strong coordination and complexation of Cu^{2+} ions with the aza-crown ether sites.

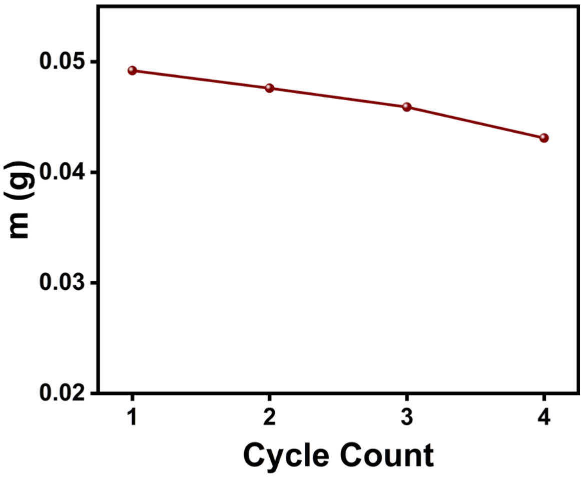

Figure S2. Mass loss of ACSA-4 before and after four regeneration cycles.

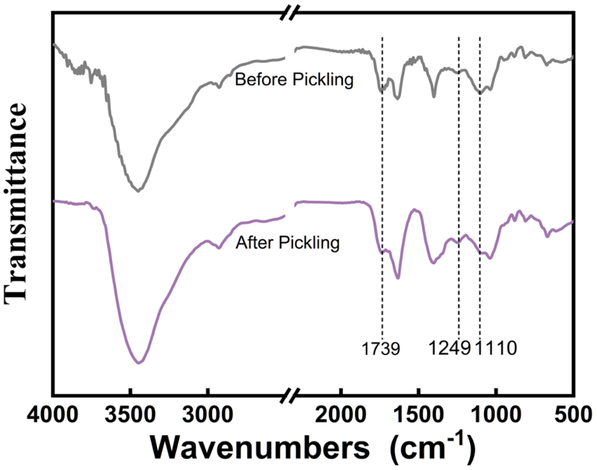

Figure S3. FTIR spectra of ACSA-4 before and after four regeneration cycles.

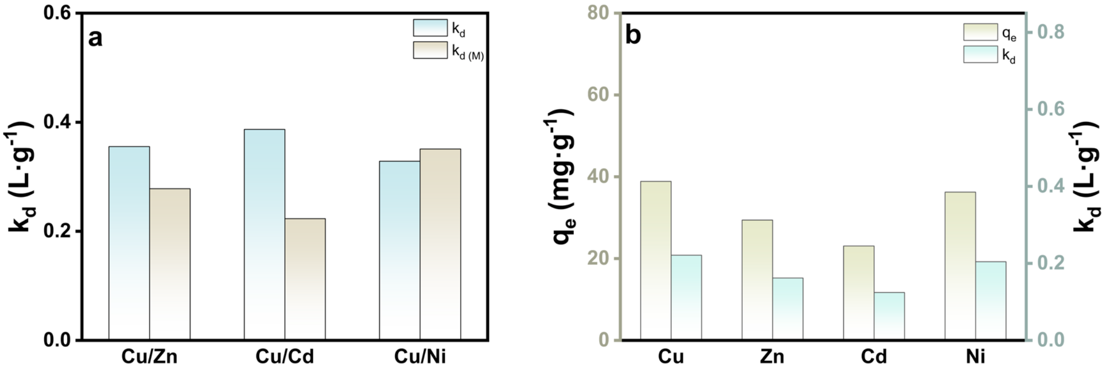

Figure S4. (a) Coexistence system of Cu^{2+} with a single metal ion using NIACSA; (b)

Coexistence system of Cu^{2+} with multiple metal-ions using NIACSA

Table S2 Effect of salt type on Cu^{2+} adsorption capacity

Salt	q_e (mg·g^{-1})
$Cu(NO)_3 \cdot 5H_20$	67.62
$CuCl_2 \cdot 2H_20$	65.17

Reference

1. Börjesson, M.; Larsson, A.; Westman, G.; Ström, A. Periodate Oxidation of Xylan-Based Hemicelluloses and Its Effect on Their Thermal Properties. *Carbohydrate Polymers* **2018**, *202*, 280–287, doi:10.1016/j.carbpol.2018.08.110.
